# *In vivo* Experience With NRT to Increase Adherence and Smoking Abstinence Among Individuals in the Criminal Legal System: Study Protocol for a Randomized Clinical Trial

**DOI:** 10.3389/fpsyt.2022.886680

**Published:** 2022-06-21

**Authors:** Elizabeth S. Hawes, Sofía Mildrum Chana, Alexandra Faust, Julianne C. Baker, Peter S. Hendricks, Andres Azuero, Adrienne C. Lahti, Matthew J. Carpenter, Karen L. Cropsey

**Affiliations:** ^1^Department of Psychiatry and Behavioral Neurobiology, School of Medicine, University of Alabama at Birmingham, Birmingham, AL, United States; ^2^Department of Health Behavior, School of Public Health, University of Alabama at Birmingham, Birmingham, AL, United States; ^3^Department of Nursing, Family, Community and Health Systems, University of Alabama at Birmingham, Birmingham, AL, United States; ^4^Department of Psychiatry and Behavioral Sciences, Medical University of South Carolina, Charleston, SC, United States

**Keywords:** adherence, tobacco, nicotine replacement therapy (NRT), criminal legal, smoking

## Abstract

**Background:**

While tobacco use among individuals involved in the criminal legal system remains 3–4 times higher than the general population, few interventions have been targeted for this population to aid in smoking cessation. Nicotine replacement therapy (NRT) is a relatively effective and accessible smoking cessation aid; however, individuals frequently stop use of NRT early due to side effects and misperceptions about the products. The present study aims to address low medication adherence by examining the efficacy of an “*in vivo*” NRT sampling experience in individuals under community criminal legal supervision.

**Methods:**

Following recruitment through community legal outlets, participants (*N* = 517) are randomized to either an “*in vivo* NRT sampling” group or a standard smoking cessation behavioral counseling group. The *in vivo* group uses NRT in session and discusses perceptions and experiences of using NRT in real time while the standard smoking cessation counseling group receives four sessions of standard behavioral smoking cessation counseling. Both groups receive four intervention sessions and 12 weeks of NRT following the intervention. The 6-month post-intervention primary outcome measures are smoking point-prevalence abstinence and medication adherence.

**Conclusion:**

This is a novel smoking cessation intervention specifically aimed at increasing NRT adherence and smoking cessation among those involved in the criminal legal system, a group of individuals with high smoking rates and low rates of pharmacotherapy use. If proven effective, the present treatment could be a novel intervention to implement in criminal legal settings given the minimal requirement of resources and training.

This trial is registered with www.clinicaltrials.gov-NCT02938403

## Introduction

Tobacco use remains the leading preventable cause of death and disability in the United States (1). While smoking prevalence has declined to about 14% in 2019 among the general population (2), tobacco use is more than 3–4 times as common among individuals with criminal legal (CL) involvement [i.e., people who have been in jail or prison, on probation/parole, or arrested; (3–7)] (estimated prevalence of 50% to 83%) (8). Individuals in the American CL system who smoke are generally younger at initiation, smoke more cigarettes per day, are 31% more likely to screen positive for nicotine dependence (3), and have high rates of other comorbidities (9, 10). The high rates of smoking among individuals in the CL system suggests that public health messages and interventions have been largely ineffective or not adequately disseminated to this population (5). Additionally, many prisons (for incarcerations exceeding 1 year) and jails (for incarcerations no more than 1 year) have now banned smoking in their facilities (11) and many people who are incarcerated relapse following release (12). However, effective provision of evidence-based interventions for this population offers great public health significance (3, 4, 9, 10, 13), particularly given the health and related risks of smoking upon release. Individuals under community corrections supervision (i.e., probation or parole) represent the majority of the CL population (69%), (14) but have reduced healthcare access due to a lack of health insurance and poverty (15, 16). Since individuals under community supervision are required to have regular contact with CL monitoring agencies, providing smoking cessation services at this point of contact represents an untapped strategy for this under-resourced population who need services and could be routinely treated while under monitoring (17).

A small number of smoking cessation intervention studies have been conducted with the CL population (9, 18). In one, nicotine replacement therapy (NRT) combined with group therapy was provided to a sample of incarcerated women (*N* = 250). Importantly, adherence to NRT was generally low (43% adherent), though it was significantly related to abstinence (10). These results established the initial efficacy of providing NRT for smoking cessation to those in the CL system (10, 19). As mentioned earlier, smoking is now banned in most jails and prisons in the U.S. Additionally, forced abstinence in these smoke-free environments is not enough to maintain abstinence post-release (20). Therefore, interventions targeting the broader CL system are primarily needed as most individuals held in jail are not incarcerated long enough for cessation efforts to be implemented or for prolonged abstinence to occur (21). Unfortunately, few trials have specifically targeted individuals under community corrections supervision to date (22, 23).

Medication adherence can more than triple rates of cessation (24–27). However, medication adherence is particularly low among individuals from under-resourced communities due to negative perceptions of the healthcare system, including less trust in medical providers, lower belief about the efficacy of medication, difficulty accessing services, high costs, and lower health literacy (28–32). Interventions to improve medication adherence in these populations have been identified as the best way to reduce health disparities over other targets such as equalizing access to healthcare or reducing provider discrimination (33, 34). Adherence to smoking cessation pharmacotherapies generally and NRT specifically are similarly poor as most people do not use medications when attempting to quit smoking (35), and among individuals who do use pharmacotherapies, about 69% stop using them prematurely (36). Although brief psychoeducation can improve attitudes toward NRT (37, 38) as well as increase intentions for future use (39), studies measuring behavioral changes (e.g., cessation) did not find psychoeducation alone to be effective (38, 40). This suggests that more hands-on experience, such as trying the cessation medication in the presence of an interventionist, may be necessary to increase medication adherence and subsequent abstinence. This gives the interventionist the opportunity to address any questions or concerns that come up, in real time, rather than asking about the person's experiences trying the medication on their own, when they might have a hard time recalling specific details.

At least two clinical trials have examined NRT sampling and Practice Quit Attempts (PQAs) to increase NRT use and subsequent cessation among outpatient smokers (41, 42). In both, the distribution of and general (i.e., unguided) encouragement to use NRT samples produced positive change in process measures as well as actual cessation. While NRT samples were provided for PQAs, this approach relied on the participant to use the sample on their own without in-session support. Other studies have investigated a more structured sampling experience, providing NRT for in-session sampling, which also led to improved perceptions of medication compared to psychoeducation alone; however, these studies did not investigate subsequent cessation (43, 44). It is possible that providing a guided sampling paradigm of trying NRT samples could also increase adherence to NRT and further promote cessation efforts. Support for this theory is found in exposure therapy whereby exposing a person to an avoided and/or feared but benign situation brings about reduced anxiety when no negative consequences occur (45). Guided, in-session sampling of NRT is particularly well suited to the CL setting, where smokers are available for sustained and structured cessation support. Furthermore, many people in the CL system and other underserved populations are more distrustful of the medical field due to the long history of not having access to healthcare, systemic racism in medical systems, and/or being taken advantage of (8, 46, 47), which decreases the likelihood that they would try cessation medications and ask for help in quitting smoking. In our study, the availability of an interventionist to address side effects in real time and reassure participants that such side effects are normal and expected might make this population more at ease about trying these medications and sticking to them. In addition, given the minimal training and expertise required by the interventionist, the present intervention is especially suitable for these settings as well as other under-resourced environments (48).

Our novel intervention is designed to provide in-session sampling of NRT to increase long-term adherence and cessation. An in-session experience with NRT is a critical aspect of this approach, as it is direct medication experience that appears to be most strongly associated with adherence and subsequent meaningful clinical outcomes [i.e., smoking abstinence; (41)]. The present article discusses the innovative design of the NRT exposure intervention used in our ongoing clinical trial for outpatients in the CL system (NCT02938403). The trial specifically examines the impact of providing NRT in real time with an interventionist (hereafter referred to as “*in vivo*”) sampling to increase later NRT adherence and smoking cessation as compared to a standard smoking cessation counseling group.

## Methods and Analysis

### Study Design and Hypotheses

Participants are randomized to one of two conditions (1:1) testing four 30-min sessions delivered over 4 weeks. A blocked randomization procedure with random blocks sizes 10 and 20 was used to generate the randomization list. The *in vivo* group receives in session NRT sampling with a focus on expectancies for medication and experience using the medication in session. The counseling group receives standard smoking cessation behavioral counseling. All participants receive NRT for 13 weeks with additional nonintervention follow-ups at 1-, 3-, and 6-months post-intervention. Thus, all participants receive almost the same level of evidence-based medication (*in vivo* group receives an additional 2 weeks during sessions 1 and 2); the only differences are the process of introducing it (guided sampling vs. not) and the difference in behavioral sessions (e.g., focus on experience with medication vs. standard smoking cessation behavioral strategies). Specifically, the patch and lozenge were chosen, and the dosage and duration of use were based on standard of care practices (49, 50). These products were chosen because the patch provides a steady dose of nicotine throughout the day while the lozenge is a short-acting NRT, which can help curb cravings in the moment (51). Nicotine gum is another over-the-counter, short-acting option, however using gum requires dentation. Unlike the nicotine inhaler and nasal spray, the patch and lozenge do not require a prescription (44). All procedures are approved by the institutional review board at the University of Alabama at Birmingham (UAB). It is expected that individuals who experience the effects of the medication during sessions will have increased adherence and cessation relative to participants who receive standard smoking cessation counseling.

Importantly, sessions began in person for the first 364 participants, but were changed to primarily remote sessions as COVID-19 precautions were put in place, with 273 participants completing a combination of in-person sessions and some remote sessions. After 6/1/20, the study procedures were modified so that participants are now required to attend an in-person baseline visit with the remaining nine appointments conducted remotely. Participants complete the sessions (*in vivo* or standard counseling) over the phone with study staff and complete all study measures *via* email, text, or verbally over the phone.

### Participants

Participants are recruited from the University of Alabama at Birmingham (UAB) Substance Abuse programs including Beacon Addiction Treatment Center (BATC), Treatment Alternatives for Safer Communities (TASC), Court Referral (CRO) Program, drug court, mental health court, community corrections, etc. with flyers posted in relevant locations and *via* snowball recruitment. Interested participants are encouraged to call or email the study team to complete eligibility screening. All study related activities are conducted by research staff only. The recruitment goal for this study is 517 smokers currently under community corrections supervision (not incarcerated). Participants who complete all study appointments receive $440 in compensation.

Potential study participants are phone screened and must be (a) under community criminal legal supervision or will be on probation or parole over the next 6 months, (b) smoking at least 5 cigarettes/day for the past year (c) 18 years of age or older, (d) able to read and speak English, (e) able to provide contact information for at least 2 people if we cannot reach the participant (f) living in an unrestricted environment that allows smoking, (g) able to access a smartphone or a personal email address. Participants must not (h) be pregnant or breastfeeding, (i) have a cognitive impairment or untreated mental illness that interferes with informed consent (based on the judgment of the research assistant if the participant is not responding appropriately or gives any indication that they are not understanding the study), (j) have experienced (within 6 months) post-myocardial infarction or untreated severe angina, (k) have a known sensitivity to NRT or adhesive products (l) exclusively use other tobacco products (e.g., cigars, e-cigarettes; although concurrent use of other tobacco products was not an exclusion criterion), or (m) be currently receiving treatment to quit smoking. It is not an eligibility requirement that participants be motivated to quit smoking. The cutoff of five cigarettes/day was chosen based on the logic that we do not want to enroll people who are light smokers or nondaily smokers for a treatment study, given that the intervention includes use of NRT. Five cigarettes/day is commonly used as a cutoff in many other smoking treatment research studies (52–54).

Once the participants are phone screened eligible, they are invited for an in-person consent and smoking is confirmed *via* an expired Carbon Monoxide (CO) >10 ppm as well as a positive urine cotinine test. Following these final inclusion procedures, they are asked to complete survey measures as part of this baseline appointment. After completion of the baseline procedures, participants are then randomized into the intervention or smoking cessation counseling group. We opted not to include stratification variables given the large sample size in the study. As shown in Table 1, side effects are assessed at every time point the participant is expected to be using the NRT. This 37-item questionnaire asks about the most common side effects from using NRT, such as nausea, skin irritation, headaches, etc. All serious adverse events or moderate/severe adverse events are reported to the principal investigator or co-investigator immediately for further guidance. All adverse events will be documented in the research record. Further, all adverse events will be compiled and reported on an annual basis to the IRB and DSMB, as well as NIDA at the conclusion of the study.

**Table 1:** Study assessment schedule.

	**BL**	**RANDOMIZATION**	**Post-Randomization Assessment Schedule**							
**Measures or Procedures**	**Day 0 $20**		**S1 $20**	**S2 $20**	**S3 $20**	**S4 $20**	**WK8 $40**	**WK 12 $40**	**M1 FU $40**	**M3 FU $40**	**M6 FU $40**
Screening Questionnaire	X										
MINI International Neuropsychiatry Interview	X										
Addiction Severity Index-Lite	X										
Everyday Discrimination Scale	X										
Functional Social Support Questionnaire	X					X	X	X	X	X	X
Perceived Stress Scale−10 Item	X		X	X	X	X	X	X	X	X	X
Smoking History	X										
Treatment Interest	X		X	X	X	X	X	X			
Thoughts About Abstinence	X		X	X	X	X	X	X	X	X	X
Smoking Abstinence Questionnaire	X							X			
Abstinence-Related Motivational Engagement	X		X	X	X	X	X	X	X	X	X
Attitudes about Nicotine Replacement Therapy	X					X	X	X	X	X	X
Medication Adherence Questionnaire (MAQ-8)			X	X	X	X	X	X	X		
Fagerström Test for Nicotine Dependence	X										
Wisc Inventory of Smoking Dependence Motives	X										
Questionnaire of Smoking Urges	X		X^1^	X^1^	X^1^	X^1^	X	X	X	X	X
Minnesota Nicotine Withdrawal Scale	X		X^1^	X^1^	X^1^	X^1^	X	X	X	X	X
Cotinine Test	X										
Urine drug screen	X							X			
Pregnancy test	X					X	X	X			
Weekly Smoking Behavior	X		X	X	X	X	X	X	X	X	X
NRT Adherence				X	X	X	X	X	X		
Treatment Satisfaction Survey						X	X	X			X
Perceived Risks of Nicotine Replacement Scale	X		X	X	X	X	X	X			
*In-vivo* Treatment Expectations	X		X	X	X	X	X	X	X	X	X
Credibility Expectancy Questionnaire (CEQ)			X				X	X	X	X	X
Alliance Questionnaire						X					
Carbon Monoxide Test (CO-iCO smokerlyzer)	X		X	X	X	X	X	X	X	X	X
Carbon Monoxide Test (CO-Vitalograph)	X										
Side Effect Scale	X		X	X	X	X	X	X	X		

*BL, Baseline Assessment; S, session; WK, week; M, month; ^1^All participants will complete these surveys twice (at the beginning and end of session) during sessions 1–4*.

### *In vivo* Intervention Group

Participants in the *in vivo* intervention group receive NRT products and counseling focused on their experience of using NRT, including positive experiences, side effects, and smoking cessation expectancies. Intervention participants are instructed to go as long as possible without smoking prior to each *in vivo* session, although participants are not excluded from the study for recent smoking. The rationale for instructing participants to abstain before each session is to demonstrate the effect of NRT for relief of withdrawal symptoms. All sessions are approximately 30 min long and are conducted individually by bachelor's-level research assistants trained by the principal investigator, who is an expert in tobacco treatment, in the *in vivo* and standard smoking cessation counseling interventions.

All participants (regardless of group) complete the Minnesota Nicotine Withdrawal Scale (MNWS) and Questionnaire of Smoking Urges (QSU) at the beginning of and end of each session. The intervention at each session focuses on their current experience of the product in real time and prior experiences with these NRT products. Feedback is provided on how their craving and withdrawal changes with use of their NRT product(s) (e.g., “your total craving score was 29 and now it is 10 after using the lozenge”). Then, each participant is given instructions on how to use the NRT product(s) between sessions. We solicit positive (e.g., “the patch helps with cravings”) as well as negative perceptions of the NRT products (e.g., “the patch makes my arm itch”) as the participant samples each product. Participants discuss any side effects they experience after using the product in session and their expectations for the effectiveness of the product for smoking cessation. Safety and efficacy results specific to the product are reviewed with the participant in session. Participants are also encouraged to use the NRT products for practice quit attempts (PQAs) between sessions, which are formally assessed in the questionnaires at the beginning of each session. While a more formal quit attempt is encouraged between sessions three and four, there are no consequences if the participant does not remain abstinent for that session. Since COVID protocols were put in place, the research assistants now provide all NRT products during the baseline appointment and are notifying participants when to start sampling the NRT products prior to their intervention phone appointment.

At session one, participants try the nicotine patch in session under direction of the therapist. Prior to patch placement, they are asked about their perceptions and prior experiences about the nicotine patch. Following patch placement, they are asked about their current experience with the patch (e.g., things they notice, positives as well as negatives, etc.). After this discussion, participants are given seven patches to use for the upcoming week outside of session, with the dose based on number of cigarettes reported at baseline. At session two, participants try a nicotine lozenge following the same procedures as above. At the end of the session, they are given three tubes of 27 count mini lozenges (2 mg). Participants are encouraged to use 8–10 lozenges a day (maximum of 20). At session three, participants try both the patch and lozenge concurrently in session and follow the same procedures in the previous session. They are then given seven patches and three tubes of 27 mini lozenges (2 mg) and asked to set a quit date and make a quit attempt prior to session four. Finally, at session four participants are given 28 patches and 12 tubes of mini lozenges for cessation attempts before their first 1-month follow-up session. For specific session information, see Table 2.

**Table 2:** Session information by group.

**Session #**	***In vivo* Intervention: NRT Products**	**Smoking Cessation Counseling Group**
1	Patch (~30 min prior to session), dose is based on CPD at baseline, complete pre- and post-administration withdrawal and craving measures, explore expectancies and side effects of patch use, given 1 week supply for PQAs	Covers benefits of quitting, eliciting social support from family/friends, goals and reasons for quitting and solicit feelings about preparing to quit
2	Lozenge (~15 min in session use), dose is based on time of first cigarette after waking at baseline, complete pre- and post-administration withdrawal and craving measures, reflect on experience using patch prior week, explore expectancies and side effects of lozenge use, given 1 week supply for PQAs	Focuses on the behavioral factors associated with smoking and the physical symptoms related to nicotine withdrawal. Discuss strategies to cope with craving and withdrawal symptoms
3	Patch and Lozenge (1 week supply of patch and lozenge), complete pre- and post-administration withdrawal and craving measures, reflect on experience using lozenge prior week, explore expectancies and side effects of combination NRT use, assisted in setting a quit day before Session 4	Focus on problem solving strategies to use for successful abstinence including letting friends/family know about quitting, relaxation strategies, soliciting support for quitting, and stimulus control. Received 1 week supply of patch and lozenge and set quit date before Session 4
4	Reflect on experiences with combination NRT prior week. Review problems encountered during quit attempt and solicit solutions. Provided with 4 weeks of patch and lozenge to use for cessation	Focus on gains made during the intervention and discuss the threat of relapse. Discussed problems encountered during quit attempt and solicited solutions. Provided with 4 weeks of patch and lozenge to use for cessation.

*When enrollment resumed with new COVID procedures, participants in both groups received all their NRT during the baseline appointment and were subsequently instructed on when and how to use it depending on group assignment*.

### Smoking Cessation Counseling Group

Participants in the smoking cessation counseling group receive behavioral smoking cessation counseling based on best practice guidelines (26). This same four-session counseling intervention was used in our previous smoking cessation intervention with participants in the CL system and was found to be acceptable and feasible with this population (48). While this intervention does not focus exclusively on use of NRT, proper NRT use is included as part of any standard behavioral intervention for smoking cessation and is recommended as a best practice guideline when combined with counseling (26). Combination NRT (patch and lozenge) was chosen for this group based on the knowledge that using both a long-acting and short-acting NRT product is the most effective way of using NRT to aid in cessation attempts (51). However, NRT is not used *in vivo* during counseling sessions and participants are not asked to abstain prior to their appointments. Participants are given a supply of seven patches (dose based on smoking reported at baseline) and three tubes of 27 lozenges each (2 mg) to use after their third session. A quit attempt is encouraged between sessions three and four, however there are no consequences if the participant does not make an attempt. At session four, participants are given the same amount of NRT as the *in vivo* group, 28 patches and 12 tubes of lozenges for smoking cessation before their first follow-up. Similar to *in vivo* participants, counseling participants complete the MNWS and QSU before and after each session but are not given any feedback on these surveys. All sessions are conducted by a research assistant trained in the intervention by the principal investigator, who is a clinical psychologist with research and clinical experience in both tobacco treatment and training other clinicians. Sessions are ~30 min in length matched to the *in vivo* counseling length.

### Therapist Training and Fidelity

Therapists for the study are trained on delivering both the *in vivo* and standard smoking cessation counseling protocols by the principal investigator of the study. The one-day training session includes reviewing the importance of smoking cessation, behavioral strategies for quitting, etc. The manual is highly structured to facilitate adherence to the intervention. When counselors covered the topics with at least 90% accuracy during practice, they were able to take study patients on their own. However, if they did not reach the 90% mark, they underwent further training and practice. While it is impossible to blind the intervening therapists to the current behavioral treatment they are delivering, the intervening therapist does not complete the follow-up sessions for the participants they treat. The follow-up assessor remains blind to the intervention delivered to participants. In addition, since most participants are able to complete the measures independently on a surface pro tablet or remotely through REDCap, no opportunity is present for the therapist to influence self-reported changes.

Sessions 1 through 4 are audio recorded by study staff for fidelity checks and 20% of all recordings are reviewed using fidelity worksheets to assess the session therapist adherence to the session. The other staff members trained in the intervention complete the fidelity worksheets for each other, so that no one completes them for sessions they conduct themselves. On each worksheet, there are specified topics that the therapist covers based on the therapist manuals. The reviewer indicates (yes/no) on the worksheet whether the therapist covered the session topics while listening to the audio recording for the session. Staff were trained by the PI using the worksheet to ensure coverage of specific items and topics. Therapists are required to score 90% or higher on the session otherwise they undergo additional training on the counseling interventions. Supervision was given during weekly meetings with staff and the principal investigator.

### Follow-Up Procedures

**Figure 1:**
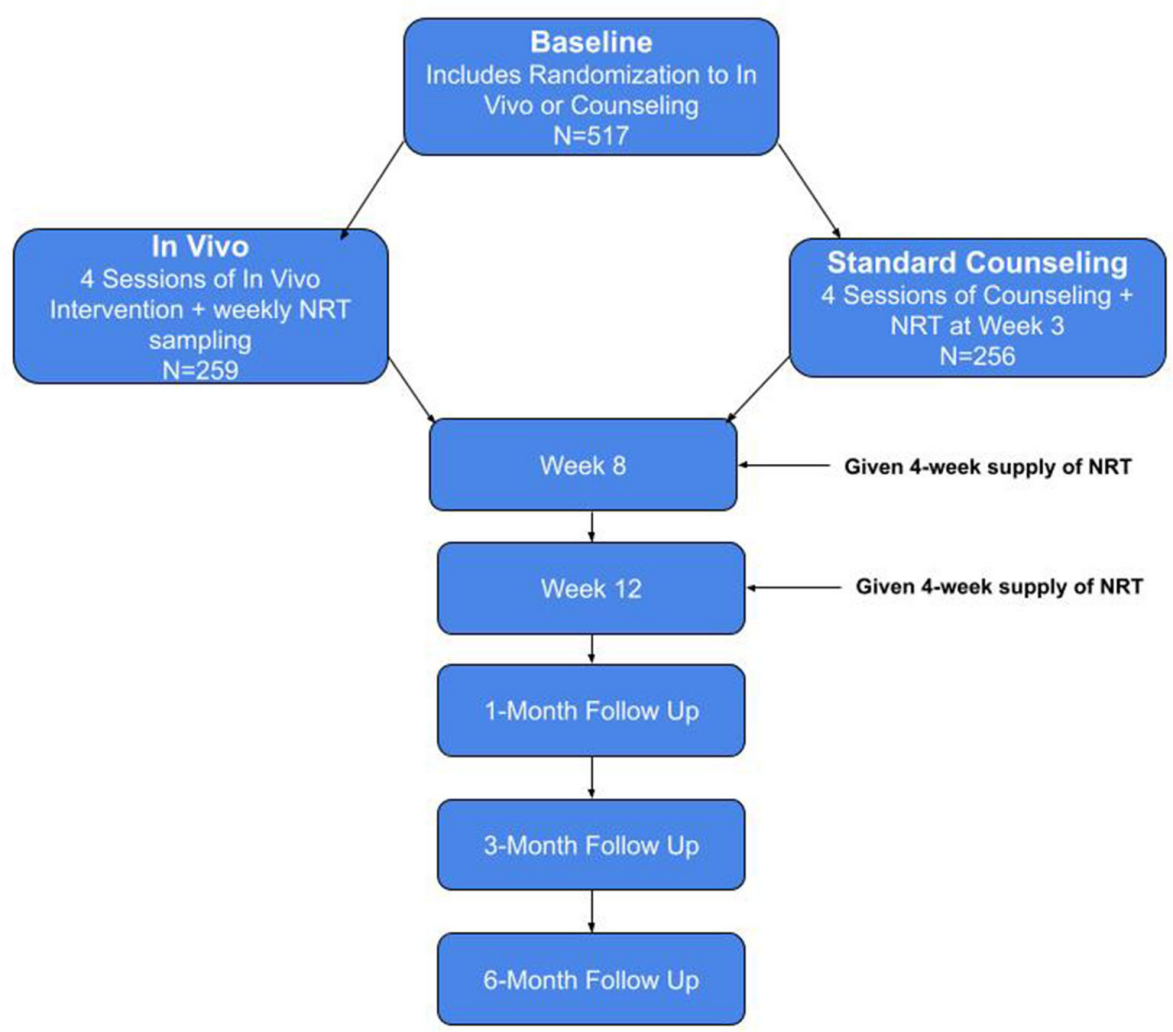
Study flow chart.

Following the four sessions for both groups, participants complete six brief check-ins to confirm their contact information (weeks 6, 10, 14, 20, 28, and 32) and five follow-up visits (weeks 8 and 12, months 1, 3, and 6), as shown in Figure 1. At each follow-up visit, participants complete questionnaires sent through an email/text link or over the phone with study staff. The participant also provides a carbon monoxide reading.

Prior to social distancing measures due to the COVID pandemic, CO was tested at all visits using the Vitalograph CO monitor. After start of COVID distancing measures, participants began being tested at baseline using both the Vitalograph CO monitor and the iCO Smokerlyzer monitor and then are given the iCO to use remotely. The Covita iCO Smokerlyzer is an individual CO monitor that connects to the participant's phone *via* the headphone jack and utilizes a phone application (iCO Smokerlyzer) to measure the participant's CO. The application instructs the participant how to complete the CO testing, asks them how many cigarettes per day (CPD) they are smoking, along with how soon they start smoking after waking up, and provides feedback to the participant about their CO level (e.g., Heavy smoker, Moderate smoker, etc.). Participants share their CO reading results with study staff *via* email directly from the iCO app, which allows study staff to continue to remotely verify smoking status in participants.

At weeks 8 and 12, participants are given instructions and reminders about the remaining NRT (4-week supply at both time points) and are reminded to track their tobacco and NRT use with study calendars until the Month 1 follow-up. At all follow-up visits until Month 6, study staff also verbally confirm pregnancy status for individuals who could become pregnant.

### Outcomes

The primary outcome variable will be smoking 7-day point prevalence abstinence confirmed by a CO ≤ 3ppm (if measured using the Vitalograph) or CO <6 ppm [if measured using the iCO Smokerlyzer; (55, 56)] at the 6-month follow-up. We will also examine abstinence across the study using the same CO-verified self-reported 7-day point prevalence abstinence. A secondary outcome includes medication adherence (defined as using >80% of doses) during the 10-week intervention period between groups. Adherence is assessed by self-report using timeline followback (TLFB) methods and prior to COVID, included verification through returned patches and blister packs of lozenges. Additional outcomes of interest include quitting across time and incidence, frequency, and duration of quit attempts. If participants are using other tobacco products, this will be reflected in the tobacco-use survey used to determine 7-day point prevalence abstinence for all products (e.g., e-cigarettes, cigars, chew/dip, etc.) as well as in the CO data for products that increase expired CO. In addition, this will be regarded as continued tobacco use and not as quit. Similarly, if participants are smoking other substances (e.g., cannabis) and are over the CO cutoff, regardless of self-report, they will be considered as smoking as we cannot separate the source of smoke through CO at follow-up. This is more conservative but given the high rates of comorbid tobacco and other drug use, particularly cannabis, it is unlikely they would stop one smoking behavior but continue the other.

### Sample Size Considerations and Data Analysis

We powered this study based on its primary Aim: intervention effects on 6-month smoking abstinence. Assuming a reference abstinence proportion of 5.8% in the smoking cessation counseling group (based on our previous bupropion trial in the CL population), using a likelihood ratio test of proportions at a significance level of 0.05, a sample size of 250 per group provides 80% power to detect a difference of 7.3% (i.e., 13.1% abstinence in the *in vivo* group, OR = 2.45).

To determine whether the abstinence rates differ over time between the two groups, a repeated measures model fitted with a generalized linear mixed-effects model or generalized estimating equations (GEE) will be used, including up to the 6-month follow-up period. This modeling approach includes a covariance structure among the repeated measurements within participants and will use all available data. If necessary, baseline covariates showing relevant baseline imbalances or associated with attrition will be included (57). Measures of effect size (e.g., Cohen's d, Cramer's V) will be used to determine baseline balance in covariates as well as the magnitude of the association between dropout and covariates. We will conduct similar analyses to evaluate differences in incidence rates of 24-h quit attempts as well as longest duration of quit attempt. To examine the secondary outcome of the project (intervention effects on medication adherence during the 10-week intervention period), we will use repeated measures modeling, as described above.

Exploratory moderation analyses will be conducted to determine whether baseline psychosocial variables such as ethnoracial identity, gender, educational attainment, and annual income, or smoking characteristics such as motivation to quit, abstinence self-efficacy, prior use of NRT, and abstinence-related expectancies moderate the relationship between intervention group and abstinence. These analyses will be conducted by fitting models with interaction terms for study group by moderator. We will also conduct mediation analyses (58, 59) to determine if factors such as medication adherence, withdrawal and craving, treatment engagement, and motivation to quit mediate the effect of intervention group on abstinence. These analyses will be conducted using path modeling to partition the intervention effect into direct and indirect. Lastly, we will examine treatment retention (% of people who were not lost to follow-up at 6-months), and engagement (# of study appointments completed) as well as therapist ratings for participants who completed study visits all in person, all remote, or a mixture of both modalities.

## Discussion

### Significance

The present intervention evaluates whether *in vivo* sampling of NRT can lead to increased smoking abstinence rates compared to standard behavioral smoking cessation treatment among individuals in the CL system. As mentioned earlier, both groups receive NRT although only the *in vivo* group receives guided and structured sampling in real time with a counselor. If the proposed hypothesis is supported, this intervention could serve as a novel strategy that improves NRT adherence in a quit attempt among individuals in the CL population. The protocol outlined above is feasible to conduct in a wide variety of settings and requires minimal training from staff. In fact, non-therapist, bachelor's-level research assistants trained on both interventions following a therapist manual are the individuals responsible for administering the intervention, underscoring the translatability of this intervention in under-resourced settings. Specifically, the findings will especially benefit individuals involved in the CL system, a population for which smoking cessation interventions are lacking and tobacco use prevalence is high. The low cost and availability of NRT in the U.S., in addition to the limited resources necessary for the intervention, make it easily implementable in community programs as well. Furthermore, the intervention could also be implemented in other settings, such as homeless shelters and hospitals, as those are opportune locations to intervene with under-resourced populations with high smoking rates (60, 61). Initially, training members of the community in this intervention would be required but in the long run, given the impact of smoking on one's health and quality of life, the benefits outweigh the initial costs. While this intervention is specific to NRT, the protocol could potentially be adapted to other smoking cessation medications with low adherence [e.g., varenicline; (62)] as well as other health conditions (e.g., diabetes management) where medication adherence is low.

### Remote Research Implications

#### General Methodology

While the present intervention was initially intended to be conducted as an in-person intervention across all study time points, recruitment and study procedures were adjusted to limit staff-participant contact given the development of the COVID-19 pandemic. Therefore, the intervention was modified to include one initial in-person baseline visit (primarily to verify smoking status and provide participants with the treatment manual, iCO monitor, and NRT products), with the remaining visits being conducted remotely. The circumstances of the pandemic led to the realization that some research procedures might benefit from the convenience and versatility of remote methods. For example, an increasing number of assessments are now being completed through platforms such as REDCap and Qualtrics, and it is plausible that entire interventions could be adapted to other remote modalities (i.e., phone calls, videoconferencing;53). In addition, consenting procedures as well as recruitment advertising can be easily performed remotely (63). Our team's adaptations for this study suggest that it is feasible to not only deliver smoking cessation interventions and counseling over the phone, but also to give smokers instructions on how to use NRT remotely, even in difficult to reach populations such as the CL population. If medication adherence and cessation outcomes are similar or better for those who complete study procedures remotely, these findings could provide support for moving to a more remote model of research. We plan to conduct a secondary data analysis to compare recruitment rate and retention between those who completed the study pre-pandemic to those post-pandemic.

#### Remote CO Collection

When the study began, carbon monoxide (CO) readings were captured using a Vitalograph CO Monitor. In order to be eligible for participation, participants' CO reading must be >10 ppm at baseline, and if their CO reading is 3 ppm or less (as measured by the Vitalograph) at follow-up assessments, they are considered abstinent from cigarettes. The study protocol was modified as in-person recruitment resumed. Since the new procedures included only one in-person visit, adjustments were necessary so study staff could continue to monitor the participant's smoking status remotely. Study staff continue to use the Vitalograph at the in-person baseline visit as well as train participants to use a take-home device, the Covita iCO Smokerlyzer as mentioned earlier. Study staff assists the participants with downloading the app, setting up an account, and walking through the procedures to complete a breath sample using the participants' own phones at the baseline appointment. The recommended cutoff to determine abstinence with this device is <6 ppm (55, 56).

#### Therapeutic Interaction

The central premise of the presented *in vivo* exposure is to have a trained bachelor's level therapist present while the participants try the cessation medication and explore expectancies and their experiences with using NRT. As previously mentioned, the therapist encourages participants to provide feedback (both positive and negative) on the use/experience of the medication, helps address any possible side effects, and provides additional product information. It is possible that this therapeutic interaction may be affected by the modality of the interaction (64, 65) (i.e., in person vs. remote). While therapeutic alliance may be comparable in remote vs. in-person sessions, each modality has specific challenges. For example, in-person sessions bring with them the logistical challenges of traveling to an in-person appointment, while a barrier to remote sessions could be limited access to technology or low technology literacy.

### Limitations and Strengths

While the intervention described presents numerous benefits and provides novel contributions to the field, several limitations should be noted. One potential limitation is that the intervention is not particularly tailored to the needs of the CL population. While it cannot be overlooked that those in the CL system have many important needs (e.g., financial, housing, and employment assistance), smoking cessation interventions remain extremely important (3, 4) especially given the negative health effects of smoking. Though these structural barriers cannot be fixed with a single intervention, evidence suggests that medication adherence is a critical target for increasing abstinence (33, 34) and that experience with smoking pharmacotherapy may be an important way to improve medication adherence in this population (23). With limited access to healthcare resources for extended periods of time, implementing a smoking cessation treatment plan into CL operations gives individuals the otherwise difficult-to-access opportunity to get help quitting smoking, which will ultimately lead to better health outcomes. It can also be argued that another weakness of the study is that the treatment is not imbedded in the community corrections system. However, this is a limitation inherent in most research that tests the efficacy of a new approach prior to expanding into more implementation science research, in this case with the community corrections staff.

An additional limitation of the present intervention could be that it is not intensive enough to promote a change in smoking behavior. A sampling intervention such as this may not provide the intensive and prolonged treatment that is necessary for a chronic relapsing condition such as smoking (66). However, time and resource-heavy interventions are unlikely to be implemented in busy clinical or low-resource settings. The proposed intervention was designed to be brief and simple enough to be implemented in busy settings by non-therapist providers but still intensive and targeted to encourage behavior change. While the PI is not a Tobacco Treatment Specialist (TTS) and the staff were not sent for specific TTS training, they are a clinical psychologist with research and clinical experience in both treating and training other clinicians about tobacco treatment,. Furthermore, RA level staff completed the counseling treatment fidelity ratings rather than a clinical supervisor or an outside clinician. Finally, as previously mentioned, intervention procedures were adapted in order to reduce staff-participant contact as the COVID-19 pandemic evolved. While necessary, these adjustments (e.g., primarily conducting remote sessions) could negatively affect the *in vivo* experience and impact the therapeutic interaction. Additionally, another potential issue in the study is the ability to retain participants until study end. Oftentimes people in the CL population have unpredictable lives, where unstable housing situations, cell phone access, transportation, etc. can impact participants' ability to adhere to the study protocol (67). Missed appointments are expected to be similar to our previous study (25–30% missed appointments at any point up to 6 months post intervention). The analytical approach will use all available data adjusted for characteristics relevantly associated with attrition, if any, and thus decreasing potential bias from missing data (57).

Nevertheless, the integrity of the intervention is generally maintained through phone calls with participants, where they can share feedback and discuss questions with the study staff. Further, with the use of the iCO, biochemical verification of smoking status is maintained at all study visits, a significant strength. A final strength of this study was the comparison group (four 30-min behavioral smoking cessation counseling sessions); thus, if the *in vivo* intervention shows stronger cessation results over the current standard, this will provide an important advance in smoking cessation treatment.

## Conclusion

The presented intervention seeks to improve NRT adherence and smoking cessation over current best practice guidelines. The intervention is delivered onsite where individuals commonly attend to check in for CL supervision. Furthermore, the intervention was adapted due to COVID-19 restrictions by pivoting to remote methods. As such, the current study presents a compelling and innovative contribution to the literature with implications for smoking cessation, NRT adherence, and remote methodologies.

## Ethics Statement

The studies involving human participants were reviewed and approved by UAB Office of Institutional Review Board (University of Alabama at Birmingham). The patients/participants provided their written informed consent to participate in this study.

## Author Contributions

PH, AA, AL, MC, and KC designed and planned the study. AA performed the calculations and verified the analytical methods. AF and JB ran the study visits. EH, SMC, AF, JB, and KC wrote the manuscript with input from all authors.

## Funding

This work was supported by the National Institute on Drug Abuse (R01DA039678) awarded to Karen Cropsey, Psy.D.

## Conflict of Interest

MC has received consulting honoraria from Pfizer. PH is on the scientific advisory board of Bright Minds Biosciences Ltd., Eleusis Benefit Corporation, and Reset Pharmaceuticals Inc. The remaining authors declare that the research was conducted in the absence of any commercial or financial relationships that could be construed as a potential conflict of interest.

## Publisher's Note

All claims expressed in this article are solely those of the authors and do not necessarily represent those of their affiliated organizations, or those of the publisher, the editors and the reviewers. Any product that may be evaluated in this article, or claim that may be made by its manufacturer, is not guaranteed or endorsed by the publisher.

## References

[1] US Preventive Services Task Force. Interventions for Tobacco Smoking Cessation in Adults, Including Pregnant Persons: US Preventive Services Task Force Recommendation Statement. JAMA. (2021) 325:265–79. 10.1001/jama.2020.25019 33464343

[2] Cornelius ME. Tobacco Product Use Among Adults—United States, 2019. MMWR Morb Mortal Wkly Rep. (2020) 69:1736–42. 10.15585/mmwr.mm6946a4 33211681 PMC7676638

[3] Winkelman TNA Vickery KD Busch AM. Tobacco use among non-elderly adults with and without criminal justice involvement in the past year: United States, 2008–2016. Addict Sci Clin Pract. (2019) 14:2. 10.1186/s13722-019-0131-y 30635028 PMC6329085

[4] Ahalt C Buisker T Myers J Williams B. Smoking and smoking cessation among criminal justice-involved older adults. Tob Use Insights. (2019) 12:1179173X19833357. 10.1177/1179173X19833357 30890860 PMC6416677

[5] Spaulding AC Eldridge GD Chico CE Morisseau N Drobeniuc A Fils-Aime R . Smoking in correctional settings worldwide: prevalence, bans, and interventions. Epidemiol Rev. (2018) 40:82–95. 10.1093/epirev/mxy005 29746635 PMC5982806

[6] Kennedy SM Sharapova SR Beasley DD Hsia J. Cigarette smoking among inmates by race/ethnicity: impact of excluding african american young adult men from national prevalence estimates. Nicotine Tob Res Off J Soc Res Nicotine Tob. (2016) 18:S73–8. 10.1093/ntr/ntv157 26980867 PMC5100810

[7] Vaughn MG DeLisi M Beaver KM Perron BE Abdon A. Toward a criminal justice epidemiology: Behavioral and physical health of probationers and parolees in the United States. J Crim Justice. (2012) 40:165–73. 10.1016/j.jcrimjus.2012.03.001

[8] Binswanger IA Carson EA Krueger PM Mueller SR Steiner JF Sabol WJ. Prison tobacco control policies and deaths from smoking in United States prisons: population based retrospective analysis. The BMJ. (2014) 349:g4542. 10.1136/bmj.g4542 25097186 PMC4122735

[9] Andrade D de Kinner SA. Systematic review of health and behavioural outcomes of smoking cessation interventions in prisons. Tob Control. (2017) 26:495–501. 10.1136/tobaccocontrol-2016-053297 27798322 PMC5574402

[10] Cropsey K Eldridge G Weaver M Villalobos G Stitzer M Best A. Smoking Cessation Intervention for Female Prisoners: Addressing an Urgent Public Health Need. Am J Public Health. (2008) 98:1894–901. 10.2105/AJPH.2007.128207 18703440 PMC2636452

[11] Kauffman RM Ferketich AK Wewers ME. Tobacco policy in American prisons, 2007. Tob Control. (2008) 17:357–60. 10.1136/tc.2007.024448 18603604

[12] Frank MR Blumhagen R Weitzenkamp D Mueller SR Beaty B Min S-J . Tobacco use among people who have been in prison: relapse and factors associated with trying to quit. J Smok Cessat. (2017) 12:76–85. 10.1017/jsc.2016.3 29430256 PMC5807014

[13] Parker DR Fallone D Martin RA Stein LAR Bock B Martin SA . The relation between smoking status and medical conditions among incarcerated adults. J Addict Med. (2014) 8:90–5. 10.1097/ADM.0b013e3182a96466 24503925 PMC4077401

[14] Minton TD. Correctional Populations in the United States, 2019—Statistical Tables. Stat Tables. (2019) 15:1–15.

[15] Eldridge GD Cropsey KL. Smoking Bans and Restrictions in U.S. Prisons and Jails: Consequences for Incarcerated Women. Am J Prev Med. (2009) 37:S179–80. 10.1016/j.amepre.2009.05.009 19591759

[16] Cropsey KL Jones-Whaley S Jackson DO Hale GJ. Smoking characteristics of community corrections clients. Nicotine Tob Res Off J Soc Res Nicotine Tob. (2010) 12:53–8. 10.1093/ntr/ntp172 19996145 PMC2902910

[17] O'Connell DJ Visher CA Becker P. Linking individuals on probation to health care: a pilot randomized trial. Health Justice. (2020) 8:8. 10.1186/s40352-020-00110-w 32236788 PMC7110823

[18] Valera P Acuna N Vento I. The Preliminary Efficacy and Feasibility of Group-Based Smoking Cessation Treatment Program for Incarcerated Smokers. Am J Mens Health. (2020) 14:1557988320943357. 10.1177/1557988320943357 32705965 PMC7383630

[19] Cropsey KL Eldridge GD Weaver MF Villalobos GC Stitzer ML. Expired carbon monoxide levels in self-reported smokers and nonsmokers in prison. Nicotine Tob Res Off J Soc Res Nicotine Tob. (2006) 8:653–9. 10.1080/14622200600789684 17008192

[20] Puljević C Segan CJ. Systematic review of factors influencing smoking following release from smoke-free prisons. Nicotine Tob Res. (2019) 21:1011–20. 10.1093/ntr/nty088 29733380

[21] Reaves B Cohen T. Pretrial release of felony defendants in state courts. (2007) 18:1–18.

[22] Garver-Apgar C Young S Howard B Udochi B Morris C. Effects of a statewide tobacco cessation program among individuals involved with Arkansas community correction. J Correct Health Care Off J Natl Comm Correct Health Care. (2017) 23:259–70. 10.1177/1078345817709017 28534434

[23] Cropsey KL Clark CB Stevens EN Schiavon S Lahti AC Hendricks PS. Predictors of medication adherence and smoking cessation among smokers under community corrections supervision. Addict Behav. (2017) 65:111–7. 10.1016/j.addbeh.2016.10.010 27816035 PMC5907501

[24] Cropsey KL Clark CB Zhang X Hendricks PS Jardin BF Lahti AC. Race and medication adherence moderate cessation outcomes in criminal justice smokers. Am J Prev Med. (2015) 49:335–44. 10.1016/j.amepre.2015.03.014 26091924 PMC4546875

[25] Shiffman S Sweeney CT Ferguson SG Sembower MA Gitchell JG. Relationship between adherence to daily nicotine patch use and treatment efficacy: secondary analysis of a 10-week randomized, double-blind, placebo-controlled clinical trial simulating over-the-counter use in adult smokers. Clin Ther. (2008) 30:1852–8. 10.1016/j.clinthera.2008.09.016 19014840

[26] Panel TU DG. Treating Tobacco Use and Dependence: 2008 Update. https://www.google.com/search?q=Washington+DC&stick=H4sIAAAAAAAAAOPgE-LQz9U3SKusLFQCs4oyzLK1tLKTrfTzi9IT8zKrEksy8_NQOFYZqYkphaWJRSWpRcWLWPnCE4szMvPSS_LzdBRcnHewMu5iZ-JgAADeRvDsWwAAAA&sa=X&ved=2ahUKEwjE2tb0-Yb4AhWCsjEKHWlwAX8QmxMoAXoECE4QAw Washington, DC: US Department of Health and Human Services (2008).

[27] Raupach T Brown J Herbec A Brose L West R A. systematic review of studies assessing the association between adherence to smoking cessation medication and treatment success. Addict Abingdon Engl. (2014) 109:35–43. 10.1111/add.12319 23919621

[28] Pieh-Holder KL Callahan C Young P. Qualitative needs assessment: healthcare experiences of underserved populations in Montgomery County, Virginia, USA. Rural Remote Health. (2012) 12:1816. 10.22605/RRH1816 22812680

[29] Sewell K Andreae S Luke E Safford MM. Perceptions of and barriers to use of generic medications in a rural African American population, Alabama, 2011. Prev Chronic Dis. (2012) 9:E142. 10.5888/pcd9.120010 22935144 PMC3475503

[30] Roebuck MC Liberman JN Gemmill-Toyama M Brennan TA. Medication adherence leads to lower health care use and costs despite increased drug spending. Health Aff Proj Hope. (2011) 30:91–9. 10.1377/hlthaff.2009.1087 21209444

[31] Kiortsis DN Giral P Bruckert E Turpin G. Factors associated with low compliance with lipid-lowering drugs in hyperlipidemic patients. J Clin Pharm Ther. (2000) 25:445–51. 10.1046/j.1365-2710.2000.00315.x 11123498

[32] Cutler DM Everett W. Thinking Outside the Pillbox — Medication Adherence as a Priority for Health Care Reform. N Engl J Med. (2010) 362:1553–5. 10.1056/NEJMp1002305 20375400

[33] Simeonova E. Doctors, patients and the racial mortality gap. J Health Econ. (2013) 32:895–908. 10.1016/j.jhealeco.2013.07.002 23933996

[34] Armstrong JD Bauman A Moroney KJ Clark CB. Assessment Treatment of Addictions in Community Corrections. London, UK: IntechOpen (2021). Available from: https://www.intechopen.com/online-first/76404

[35] Centers for Disease Control and Prevention (CDC). Quitting smoking among adults—United States, 2001–2010. MMWR Morb Mortal Wkly Rep. (2011) 60:1513–9. 22071589

[36] Balmford J Borland R Hammond D Cummings KM. Adherence to and reasons for premature discontinuation from stop-smoking medications: data from the ITC Four-Country Survey. Nicotine Tob Res Off J Soc Res Nicotine Tob. (2011) 13:94–102. 10.1093/ntr/ntq215 21147894 PMC3028191

[37] Juliano LM Brandon TH. Smokers' expectancies for nicotine replacement therapy vs. cigarettes Nicotine Tob Res Off J Soc Res Nicotine Tob. (2004) 6:569–74. 10.1080/14622200410001696574 15203790

[38] Mooney ME Leventhal AM Hatsukami DK. Attitudes and knowledge about nicotine and nicotine replacement therapy. Nicotine Tob Res Off J Soc Res Nicotine Tob. (2006) 8:435–46. 10.1080/14622200600670397 16801301

[39] Ferguson SG Gitchell JG Shiffman S Sembower MA Rohay JM Allen J. Providing accurate safety information may increase a smoker's willingness to use nicotine replacement therapy as part of a quit attempt. Addict Behav. (2011) 36:713–6. 10.1016/j.addbeh.2011.02.002 21371825

[40] Hollands GJ McDermott MS Lindson-Hawley N Vogt F Farley A Aveyard P. Interventions to increase adherence to medications for tobacco dependence. Cochrane Database Syst Rev. (2015) CD009164. 10.1002/14651858.CD009164.pub2 25914910

[41] Carpenter MJ Hughes JR Gray KM Wahlquist AE Saladin ME Alberg AJ. Nicotine therapy sampling to induce quit attempts among smokers unmotivated to quit. Arch Intern Med. (2011) 171:1901–7. 10.1001/archinternmed.2011.492 22123796 PMC3372898

[42] Jardin BF Cropsey KL Wahlquist AE Gray KM Silvestri GA Cummings KM . Evaluating the effect of access to free medication to quit smoking: a clinical trial testing the role of motivation. Nicotine Tob Res Off J Soc Res Nicotine Tob. (2014) 16:992–9. 10.1093/ntr/ntu025 24610399 PMC4133568

[43] Schneider NG Koury MA Cortner C Olmstead RE Hartman N Kleinman L . Preferences among four combination nicotine treatments. Psychopharmacology (Berl). (2006) 187:476–85. 10.1007/s00213-006-0449-5 16896965

[44] Schneider NG Cortner C Justice M Gould JL Amor C Hartman N . Preferences among five nicotine treatments based on information versus sampling. Nicotine Tob Res Off J Soc Res Nicotine Tob. (2008) 10:179–86. 10.1080/14622200701767837 18188758

[45] Craske MG Treanor M Conway CC Zbozinek T Vervliet B. Maximizing exposure therapy: An inhibitory learning approach. Behav Res Ther. (2014) 58:10–23. 10.1016/j.brat.2014.04.006 24864005 PMC4114726

[46] Valera P Boyas JF Bernal C Chiongbian VB Chang Y Shelton RC . Validation of the group-based medical mistrust scale in formerly incarcerated black and Latino men. Am J Mens Health. (2018) 12:844–50. 10.1177/1557988316645152 27192716 PMC6131472

[47] Benkert R Cuevas A Thompson HS Dove-Medows E Knuckles D. Ubiquitous Yet Unclear: A Systematic Review of Medical Mistrust. Behav Med. (2019) 45:86–101. 10.1080/08964289.2019.1588220 31343961 PMC6855383

[48] Cropsey KL Hendricks PS Schiavon S Sellers A Froelich M Shelton RC . A pilot trial of In vivo NRT sampling to increase medication adherence in community corrections smokers. Addict Behav. (2017) 67:92–9. 10.1016/j.addbeh.2016.12.011 28063325

[49] Practitioners TRAC of General. Optimising nicotine replacement therapy in clinical practice. Australian Family Physician. East Melbourne, https://www.google.com/search?q=Victoria&stick=H4sIAAAAAAAAAONgVuLUz9U3SM5ILzJaxMoRlplckl-UmQgAQA9apBgAAAA&sa=X&ved=2ahUKEwi_8Lnk-ob4AhXITTABHWBMAmIQmxMoAXoECGIQAw Victoria: The Royal Australian College of general Practitioners (2022). Available from: https://www.racgp.org.au/afp/2013/may/nicotine-replacement-therapy

[50] Sweeney CT Fant RV Fagerstrom KO McGovern JF Henningfield JE. Combination nicotine replacement therapy for smoking cessation: rationale, efficacy and tolerability. CNS Drugs. (2001) 15:453–67. 10.2165/00023210-200115060-00004 11524024

[51] Ebbert JO Hays JT Hurt RD. Combination Pharmacotherapy for Stopping Smoking. Drugs. (2010) 70:643–50. 10.2165/11536100-000000000-00000 20394453 PMC3164516

[52] Valera P Malarkey S Smith N McLaughlin C. Exploring the role of telehealth: a novel approach to group-based smoking cessation treatment for men incarcerated in a rural state prison. J Telemed Telecare. (2021) 1357633X211034734. 10.1177/1357633X211034734 34524911

[53] Hugley MJ Wolford-Clevenger C Sisson ML Nguyen AT Cropsey KL. Self-initiated gradual smoking reduction among community correction smokers. Addict Behav. (2019) 93:100–3. 10.1016/j.addbeh.2019.01.028 30703663 PMC6937780

[54] Nahvi S Adams TR Ning Y Zhang C Arnsten JH. Effect of varenicline directly observed therapy versus varenicline self-administered therapy on varenicline adherence and smoking cessation in methadone-maintained smokers: a randomized controlled trial. Addiction. (2021) 116:902–13. 10.1111/add.15240 32857445 PMC7983847

[55] Tuck BM Karelitz JL Tomko RL Dahne J Cato P McClure EA. Mobile, remote, and individual focused: comparing breath carbon monoxide readings and abstinence between smartphone-enabled and stand-alone monitors. Nicotine Tob Res. (2021) 23:741–7. 10.1093/ntr/ntaa203 33022057 PMC7976935

[56] Vickerman KA Carpenter KM Miles LN Hsu JM Watt KA Brandon TH . Treatment development, implementation, and participant baseline characteristics: a randomized pilot study of a tailored quitline intervention for individuals who smoke and vape. Contemp Clin Trials Commun. (2021) 24:100845. 10.1016/j.conctc.2021.100845 34568637 PMC8449159

[57] Groenwold RH Donders AR Roes KC Harrell FE Moons KG. Dealing with missing outcomes data in randomized trials with observational studies. Am J Epidemiol. (2012) 175:210–7. 10.1093/aje/kwr302 22262640

[58] MacKinnon DP Lockwood CM Hoffman JM West SG Sheets V. A comparison of methods to test mediation and other intervening variable effects. Psychol Meth. (2002) 7:83–104. 10.1037/1082-989X.7.1.83 11928892 PMC2819363

[59] MacKinnon DP Fairchild AJ Fritz MS. Mediation analysis. Annu Rev Psychol. (2007) 58:593–614. 10.1146/annurev.psych.58.110405.085542 16968208 PMC2819368

[60] Baggett TP Lebrun-Harris LA. Rigotti NA. Homelessness, cigarette smoking, and desire to quit: results from a US National Study. Addict Abingdon Engl. (2013) 108:2009–18. 10.1111/add.12292 23834157 PMC3797258

[61] Rigotti NA Munafo MR Stead LF. Smoking cessation interventions for hospitalized smokers: a systematic review. Arch Intern Med. (2008) 168:1950–60. 10.1001/archinte.168.18.1950 18852395 PMC4500120

[62] Liberman JN Lichtenfeld MJ Galaznik A Mastey V Harnett J Zou KH . Adherence to varenicline and associated smoking cessation in a community-based patient setting. J Manag Care Pharm JMCP. (2013) 19:125–31. 10.18553/jmcp.2013.19.2.125 23461428 PMC10437765

[63] Dahne J Tomko RL McClure EA Obeid JS Carpenter MJ. Remote methods for conducting tobacco-focused clinical trials. Nicotine Tob Res Off J Soc Res Nicotine Tob. (2020) 22:2134–40. 10.1093/ntr/ntaa105 32531046 PMC7454765

[64] Probst T Haid B Schimböck W Reisinger A Gasser M Eichberger-Heckmann H . Therapeutic interventions in in-person and remote psychotherapy: Survey with psychotherapists and patients experiencing in-person and remote psychotherapy during COVID-19. Clin Psychol Psychother. (2021) 28:988–1000. 10.1002/cpp.2553 33448499 PMC8013388

[65] Simpson S Richardson L Pietrabissa G Castelnuovo G Reid C. Videotherapy and therapeutic alliance in the age of COVID-19. Clin Psychol Psychother. (2020) 10.1002/cpp.2521. 10.1002/cpp.2521 33037682 PMC7675483

[66] Hall SM Humfleet GL Muñoz RF Reus VI Prochaska JJ Robbins JA. Using extended cognitive behavioral treatment and medication to treat dependent smokers. Am J Public Health. (2011) 101:2349–56. 10.2105/AJPH.2010.300084 21653904 PMC3222443

[67] Morrison B Bowman J. What happens beyond the gate? Findings from the post-release employment study. Pract N Z Correct J. (2017) 5.

